# Development and Proof of Concept of a Miniaturized MEMS Quantum Tunneling Accelerometer Based on PtC Tips by Focused Ion Beam 3D Nano-Patterning

**DOI:** 10.3390/s21113795

**Published:** 2021-05-30

**Authors:** Michael Haub, Martin Bogner, Thomas Guenther, André Zimmermann, Hermann Sandmaier

**Affiliations:** 1 Institute for Micro Integration (IFM), University of Stuttgart, Allmandring 9b, 70569 Stuttgart, Germany; martin.bogner@ifm.uni-stuttgart.de (M.B.); thomas.guenther@ifm.uni-stuttgart.de (T.G.); andre.zimmermann@ifm.uni-stuttgart.de (A.Z.); 2 Hahn-Schickard, Allmandring 9b, 70569 Stuttgart, Germany; 3 Chair of Microsystems (MST), University of Stuttgart, Pfaffenwaldring 4F, 70569 Stuttgart, Germany; hermann.sandmaier@mst.uni-stuttgart.de

**Keywords:** tunneling effect, accelerometer, focused ion beam, fib, quantum sensor, tunneling tip

## Abstract

Most accelerometers today are based on the capacitive principle. However, further miniaturization for micro integration of those sensors leads to a poorer signal-to-noise ratio due to a small total area of the capacitor plates. Thus, other transducer principles should be taken into account to develop smaller sensors. This paper presents the development and realization of a miniaturized accelerometer based on the tunneling effect, whereas its highly sensitive effect regarding the tunneling distance is used to detect small deflections in the range of sub-nm. The spring-mass-system is manufactured by a surface micro-machining foundry process. The area of the shown polysilicon (PolySi) sensor structures has a size smaller than 100 µm × 50 µm (L × W). The tunneling electrodes are placed and patterned by a focused ion beam (FIB) and gas injection system (GIS) with MeCpPtMe_3_ as a precursor. A dual-beam system enables maximum flexibility for post-processing of the spring-mass-system and patterning of sharp tips with radii in the range of a few nm and initial distances between the electrodes of about 30–300 nm. The use of metal–organic precursor material platinum carbon (PtC) limits the tunneling currents to about 150 pA due to the high inherent resistance. The measuring range is set to 20 g. The sensitivity of the sensor signal, which depends exponentially on the electrode distance due to the tunneling effect, ranges from 0.4 pA/g at 0 g in the sensor operational point up to 20.9 pA/g at 20 g. The acceleration-equivalent thermal noise amplitude is calculated to be 2.4–3.4 mg/$\sqrt{Hz}$. Electrostatic actuators are used to lead the electrodes in distances where direct quantum tunneling occurs.

## 1. Introduction

The basic principle of an accelerometer is the deflection of a proof mass. Today’s commercial accelerometers are based on established transducer principles, such as capacitive, piezoresistive, piezoelectric, and thermal effects. In order to increase the performance and sensitivity, large proof masses and long beams are provided since noise mechanisms lead to limiting properties [1,2,3,4]. With respect to the scaling laws [5], an isometric reduction of the proof mass leads to a substantial reduction of the deflection at an applied acceleration. In the case of the piezoresistive or piezoelectric principle, the reduced deflection of the mass leads to a lower deformation and thus a significantly reduced measurement signal. In terms of the capacitive accelerometer, the reduction of the capacitor plates leads to a significant reduction of the capacitance to be measured. Since the sensor properties are mainly dependent on the signal-to-noise ratio, the miniaturization of today’s conventional methods for measuring acceleration is limited. The accelerometer MEMS size evolution of the past fifteen years shows a tremendous decrease in overall size [6]. However, a further decrease of the adequate sensing area size leads to the need of new methods and transducer principles.

### 1.1. General State-of-the-Art and Motivation

The smallest commercial accelerometer today is the MC3672 by mCube [7], built in WLCSP (wafer-level redistribution chip scale package) architecture with an overall packaging size of only 1.09 × 1.29 × 0.74 mm^3^ based on the capacitive principle. The developments by Bosch Sensortec of ultra-small capacitive accelerometers went slightly back from the BMA355 [8] with similar packing size of 1.2 × 1.5 × 0.8 mm^3^ to a size of 2 × 2 × 0.65 mm^3^ for currently available sensors (e.g., BMA456 [9]). The spring-mass-system area of small capacitive sensors amounts to around 500 × 500 µm^2^ to achieve mg resolution and 1 × 1 mm^2^ to achieve a resolution of µg [4]. To reach ng resolution, a large proof mass of several 1 × 1 mm^2^ is necessary [10]. An ultraminiaturized piezoresistive accelerometer with a flexible structure of 387 × 387 µm^2^ was published by Park et al. [11,12]. With an analytical FEM approach, Engesser et al. [13] investigated the miniaturization limits of a lateral piezoresistive sensor element related to different beam-mass configurations and the total noise limit. Song et al. [14] showed a miniaturized piezoelectric accelerometer with a size of 1.5 × 1.5 × 0.5 mm^3^ and measured a current change of some 10 pA at an applied acceleration of 1 g. Gesing et al. [15] published results about a small piezoelectric annular accelerometer with a minimum sensing area of 2 × 2 mm^2^. Furthermore, ultrasmall microelectromechanical system (MEMS) thermal accelerometers are commercially available by MEMSIC Inc. [16]. In addition to a deep cavity, the publications on this subject specify an area of the thermal-sensing element of 600 × 600 µm^2^ [17] to approximately 1 × 1 mm^2^ [18,19,20]. A less conventional accelerometer design is shown by Dong et al. [21]. They designed and realized an optomechanical accelerometer with a whisper gallery mode ring resonator and a spring-mass system at a size of around 160 × 100 µm^2^. The use of optical transducer principles is shown to be unsuitable for a harsh environment and has limited potential for the system integration [3]. Furthermore, a new approach of accelerometers miniaturization methods uses thin graphene beams and their piezoresistive characteristic [22,23]. Recently, Fan et al. [24] showed the suspension of a proof mass on a monolayer graphene beam thereby reducing the total area of the spring-mass structure in the range below 100 × 100 µm^2^. One more unconventional principle is the use of the quantum tunneling effect. A deeper insight of the surveys of tunneling accelerometers is given in Section 1.2. All these developments show the further need for the miniaturization of accelerometers. In particular, cost reduction and the integration of sensors into decreasing overall systems such as wearables play a significant role.

This study’s objective shows a novel approach by miniaturizing acceleration sensors based on the highly sensitive quantum tunneling effect. By integrating tunneling electrodes into a spring-mass-damper system, the miniaturization potential for acceleration sensors can be investigated by varying their geometric size. As shown above, scaling laws lead to an unfavorable signal-to-noise ratio when sensor structures are isometrically reduced in size and conventional methods are unsuitable for further miniaturization. Employing a more sensitive measuring principle, such as the tunneling effect, can overcome these drawbacks. This work aims to advance the miniaturization to a few 10 × 10 µm^2^. The fabrication of the sensor structure is based on the surface micro-machining (SMM) technology. The tunneling section is integrated using a dual-beam system consisting of a scanning electron microscope (SEM) and a focused ion beam (FIB) as well as a gas injection system (GIS) with a MeCpPtMe3 precursor for the deposition of metallic electrodes. With the tunneling effect, changes in distance can be detected in the sub-Angström (Å) range. While scanning tunneling microscopes and tunneling diodes as the main areas of the tunneling effect’s application have already completed the step from research to industry, sensors—based on the tunneling effect—have not yet been commercially implemented despite some thirty years of research. Previous developments of acceleration sensors utilizing the tunneling effect improved the resolution limit down to 10^−8^ g [25,26]. In this project, the focus of research and development is on miniaturizing the sensor structure in order to reduce its size, which represents a major factor for costs in IC fabrication. The area requirement of the sensor structure is to be reduced until the sensor’s resolution at high dynamics of several kHz is limited by the thermal noise of the spring-mass system in the range of today’s acceleration sensors of a few per mile of the nominal range. The technological realization of the sensor structures for a measuring range of several g is carried out with the commercial PolyMUMPs Foundry Service from MEMSCAP Inc, which is focused on proof-of-concept prototyping and allows custom surface micro-machining fabrication for low volume orders. The tunneling electrodes are integrated subsequently with a focused ion beam. Therefore, this work also focuses on micro/nano integration, i.e., the development of the deposition and structuring processes with a focused ion beam, which is integrated into the overall process as a method for the production of tunneling sections and the post-processing of the polysilicon spring-mass system. The post-processing of the SMM structures makes it possible to reduce the sensor structures’ area further.

### 1.2. Previous Work on Tunneling Accelerometers

Some work on tunneling accelerometers has already been presented in the past. The developments were based on the work of Binning and Rohrer [27], who realized the tunneling effect in a reproducible method for scanning tunneling microscopy and thus the atomic resolution of the probe. The pioneers of high-resolution tunneling accelerometers were Baski et al. [28] and Waltman and Kaiser [29]. They realized the principle of the tunneling effect in an acceleration-sensitive setup based on Binning and Rohrer’s findings [27]. The focus at the first tunneling accelerometers was on high resolution down to the range of a few ng to µg. Since the tunneling current depends strongly exponentially on the distance between the tunneling electrodes, sensors with high sensitivity (g/A) and resolution can be realized. Further work by Kenny et al. [25,26], Yeh et al. [30,31,32], and Rockstad et al. [33] showed the possibility of increasing the resolution up to 10^−8^ g and demonstrated it in a bulk micro-machining (BMM) sensor structure. Tunneling accelerometers in surface micromachining (SMM) were realized by Kubena et al. [34,35] and Patra et al. [36,37,38]. Kubena et al. showed a small sensor structure with a slightly larger size than realized in this work. The authors used the high sensitivity of the tunneling effect to realize a high dynamic range up to 10^4^ g in a forced balanced method, based on a high cantilever stiffness without an additional seismic mass. Burgner et al. [39] showed another work using silicon-on-insulator (SOI) technology. In recent years there has been a gap in the development of tunneling accelerometers, and moreover, a commercial implementation has not been carried out yet. A more recent project, “GraTa” [40], aimed to develop a graphene-based tunneling accelerometer. Further work is summarized in Table 1.

### 1.3. Quantum Physical Basics

**Figure 1:**
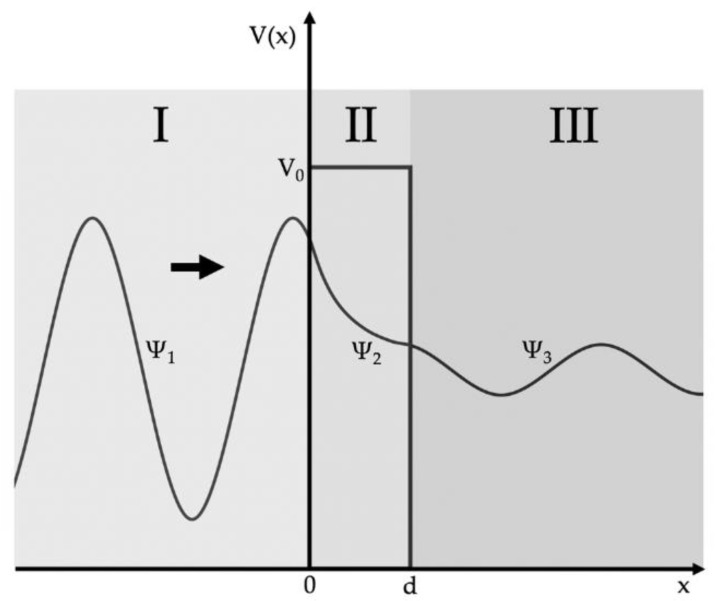
A wave arriving from the left hits a potential barrier. A part of the wave is reflected, and another part is transmitted through the barrier.

According to Erwin Schrödinger, the tunneling effect can be described by the wave function based on wave mechanics. The wave function represents the equation of motion in position or momentum space. Figure 1 shows three areas (I, II, and III) that are important when a wave hits a potential barrier. To describe the tunneling of a particle through the barrier, the wave’s behavior must be described for these three regions. The energy level of the potential barrier is higher than the energy of the particle. According to classical physics, the particle cannot overcome the barrier but is entirely reflected by it. However, the quantum tunneling effect allows the wave to transmit and appear on the other side of the barrier. In area I, a plane wave $\Psi_{1}\;$ or a particle with energy E from the left hits the potential barrier of height $V_{0}$ and width d. In area II within the potential barrier, the tunneling effect occurs through the transmission of the wave $\Psi_{2}$. Since not all of the entire wave $\Psi_{1}\;$ is transmitted, a part ($\Psi_{R}$) of it is reflected to the left. The transmitting wave $\Psi_{2}\;$ decays within the potential threshold. Section III describes the resulting wave $\Psi_{3}\;$ after escaping the potential barrier.

The time-independent Schrödinger equation is:

(1) $$\frac{-\hbar^{2}}{2\;m}\;\frac{d^{2}\Psi \left(x\right)}{dx^{2}}+V\left(x\right)\;\Psi \left(x\right)=E\;\Psi \left(x\right)$$

with ℏ as the reduced Planck’s constant, m the particle’s mass, Ψ(x)  the wave function, V(x)  the potential energy, and E the energy of the system. To solve Equation (1) and finally calculate the tunneling probability T, the function

(2) $$\Psi \left(x\right)=A\;e^{ikx}$$

is selected. For the ranges I and III, the oscillatory approach is used and the solution for the wave functions $\Psi_{1}\;$ and $\Psi_{3}\;$ with the coefficients $A_{1},B_{1},A_{3}$, and $B_{3}$ results in:

(3) $$\Psi_{1}\left(x\right)=A_{1}\;e^{ik_{1}x}+B_{1}\;e^{-ik_{1}x}$$

(4) $$\Psi_{3}\left(x\right)=A_{3}\;e^{ik_{3}x}+B_{3}\;e^{-ik_{3}x}$$

In area II, the approach for an exponentially increasing or decreasing wave function with the coefficients $A_{2}$ and $B_{2}$ is chosen and results in:

(5) $$\Psi_{2}\left(x\right)=A_{2}\;e^{k_{2}x}+B_{2}\;e^{-k_{2}x}$$

Since for x<0 and x>d the potential V(x)=0, $k_{1}$ is equal to $k_{3}$. The transitions x=0 and x=d  must be continuous and continuously differentiable to obtain a solution for the entire area. $B_{2}=0$ because a reflected wave in area III does not exist. By transforming Equation (2) $k_{n}\;$ results in:

(6) $$k_{1}=k_{3}=\frac{1}{\hbar }\;\sqrt{2\;m\;E}$$

for areas I, III and for area II in:

(7) $$k_{2}=\frac{1}{\hbar }\;\sqrt{2\;m\;\left(V_{0}-E\right)}$$

with ℏ as the reduced Planck’s constant. The transmission probability T is the quotient of the probability densities $|A_{3}{|}^{2}$ and $|A_{1}{|}^{2}$:

(8) $$T=\frac{|A_{3}{|}^{2}}{|A_{1}{|}^{2}}$$

By transformation of Equations (3)–(5), with a consideration of the continuity conditions and insertion of k the transmission probability T is calculated by:

(9) $$T=\frac{1-\frac{E}{V_{0}}}{\left(1-\frac{E}{V_{0}}\right)+\frac{V_{0}}{4E}\;sinh^{2}\left(k_{2}\;d\right)}$$

Equation (9) indicates that the transmission probability is not equal to zero at $E<V_{0}$. Thus, the tunneling effect can be defined by the fact that there is a finite probability of finding the particle on the forbidden side. Due to the potential difference, the electrons take a preferred direction. The resulting electrical current is called tunneling current. The formula of the tunneling effect was generalized by Simmons [48]. The calculation of the tunneling current between two metal electrodes can be derived by Simmons findings through the following equation [49]:

(10) $$I_{T}\propto U_{T}\;\exp\left(-2\;d\;\frac{\sqrt{2\;m_{e}\;\Phi }}{\hbar }\right)\approx U_{T}\;\exp\left(-\alpha \;d\right)$$

where $I_{T}$ is the tunneling current, d is the distance between the electrodes, $m_{e}$ is the mass of an electron, and Φ is the average barrier height. The right side of Equation (10) shows a simplification by the constant $\alpha =1.025\;eV^{-0.5}\;{Å}^{-1}$ and is the most common formula to approximate the tunneling effect by approaching two tips. With a barrier height of a few eV the equation shows a change of the tunneling current by one order of magnitude at a distance change of 1 Å [50]. This extreme distance dependency allows the observation of atomic structures. The most common application of the tunneling effect is the scanning tunneling microscope. Using fine metallic tips, the atomic structures of samples can be imaged. The tip is approached to a 1 nm probe distance and below with a piezo actuator. With other piezo actuators, the lateral axes can be traced. Due to the change in the tunneling current depending on the atoms’ position in the sample, this can be imaged by a scanning movement. The sharper the tip, the higher the lateral resolution. In the tunneling microscope, the tunneling electrodes are fixed in a way which makes sure that smaller distances can also be achieved. This is a great advantage compared to the tunneling accelerometers, which must have at least one free-moving tip for their acceleration-sensitive function. Nevertheless, the integration of tunneling electrodes to a spring-mass-system enables an enormous miniaturization of the accelerometer size. Due to the exponential dependence between the distance and the tunneling current and thus the tunneling effect’s high sensitivity, only smallest deflections are needed.

### 1.4. Sensor Principle and Operation Phases

**Figure 2:**
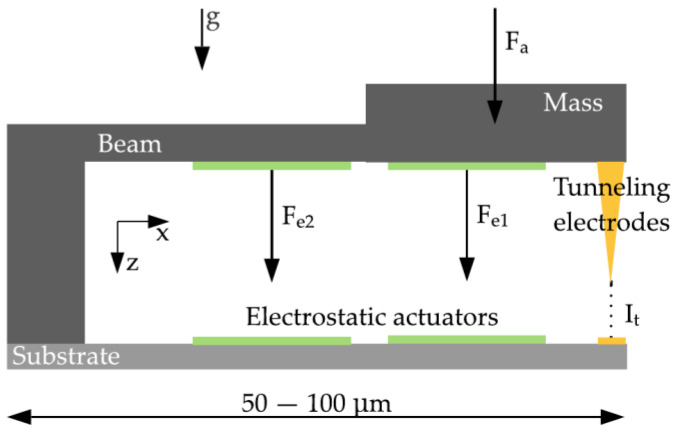
Principle of the tunneling accelerometer with its main components, the spring-mass-system, the electrostatic actuators, and the tunneling section. $F_{e}$ = electrostatic forces, $F_{a}$ = force by an applied acceleration a onto the proof mass, g = acceleration due to gravity, and $I_{t}\;$= tunneling current.

The present development’s aim is, largely independent of the resolution, the miniaturization up to the physical limits with a nominal acceleration of a few g. The sensor structure is manufactured in surface micromachining by MEMSCAP Inc. (Durham, NC, USA). The platinum (Pt) carbon (C) tunneling electrodes will be deposited subsequently in the sensor structure by a focused ion beam. This work is focused on a highly miniaturized sensor structure by integrating a tunneling tip into a typical spring-mass system. Figure 2 shows the working principle of the sensor and the sensor system components integrated directly by the PolyMUMPs process. The sensor consists of a spring-mass system with several electrodes placed on the substrate. The two electrodes, highlighted in green, are used as electrostatic actuators for pulling down the spring-mass-system (grey) to the operation point, ensuring a constant distance between the tunneling electrodes (yellow) and self-test actions. In this paper, the sensor is shown in an open-loop configuration by controlling the maximum acceleration. In this way, the spring-mass system does not overcome its maximum deflection range, and a crash of the electrodes is prevented. 

The reasonable working distance between the tunneling electrodes is in the order of a few nm. If an acceleration is applied to the spring-mass-system, the mass will be deflected, and the tunneling current changes immediately. The tunneling current depends exponentially on the tunneling electrode’s distance and is defined by (10). Since deflections in the range of sub-nm already cause changes in the tunneling current, a system of small size and consequently high stiffness can be designed. 

**Figure 3:**
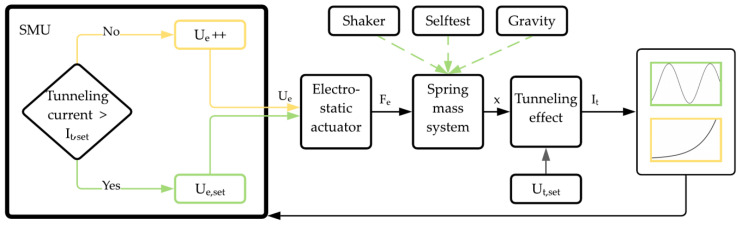
Diagram of the sensor system with the respective output variables. $U_{e}$ = actuator voltage, $F_{e}$ = electrostatic force, x = deflection, $I_{t}$ = tunneling current.

Figure 3 shows the functional structure of the running and measurement setup for the accelerometer, including essential devices. The operation of the sensor is divided into two phases. The first one (yellow) increases the voltage at the electrostatic actuator ($U_{e}$++) until an onset of a tunneling current is detected and levels at a chosen value of $I_{t,set}$. Subsequently, the sensor is in the operating mode (green) with $U_{e,set}$ being the voltage required by the operating point actuator to get the tunneling electrodes into a working distance. If a voltage by a self-test actuation ($U_{self}$), external acceleration by a shaker (△a) or the gravity field (g) affects the sensor structure, the seismic mass is deflected. A high-precision source measurement unit (SMU) is used to measure the extremely low changes in the tunneling current down to sub-pA and up to a few nA, depending on the type of excitation.

## 2. Design and Simulation

The design of the sensor structure is essentially divided into the areas of the spring-mass system, tunneling electrode, and electrostatic actuator. The design shows explicit dependencies between the shape of the tunneling electrode, the geometry of the mechanical sensor structure and the electrostatic actuators. The high sensitivity of the tunneling effect allows a high degree of miniaturization of the spring-mass structures and the associated stiffening of the system. In the present work, platinum–carbon electrodes are used. In this metal–organic material, individual platinum grains are embedded in a high-resistance diamond-like-carbon (DLC) matrix. It is known that the conduction mechanism takes place by intrinsic tunneling between the platinum grains [51,52,53,54]. Due to the carbon matrix and the additional vacuum gap, a much lower tunneling current is expected than known from highly conductive metallic tunneling electrodes as used in a scanning-tunneling microscope. Thus, a higher tunneling bias voltage is needed to provide an evaluable tunneling current. Additionally, the electrode tip radius needs to be considered in this regard, too. The radius has a significant effect on preventing a snap-in effect due to attractive forces on the tunneling section caused by electrostatic, Van der Waals (VdW), and capillary forces. The attractive forces directly limit the possible stiffness of the sensor structure in relation to the tip radius and electrode distance. Accordingly, fundamental parameters for the sensor structure were derived by analytical and numerical calculation in relation to the system stiffness and sensitivity concerning a measuring range of the sensor of several g and a resulting deflection of some Å. Since the production of the sensor structure is carried out with the PolyMUMPs foundry service, the applicable design rules for the structural models limit the geometric sizes regarding the smallest structural features/spacings on the one hand and the functional implementation regarding the SMM layers on the other hand. The subsequent testing on the implemented sensor structures shows the necessity for compliance with the smallest distances (min. 2 µm) and structural widths (min. 3 µm). With the aim to realize sensor structures of an overall size of some 10 × 10 µm^2^, these rules represent an immediate limitation for the geometric implementation. The design and layout of the electrostatic actuator are directly related to the lateral expansion of the mass, the spring, and the electrodes’ spacing (determined by the foundry service). The required actuator voltage to achieve the tunneling distance results from the initial distance of the tunneling electrodes and the geometric size of the actuator. Several structures were designed and implemented for a comprehensive and flexible investigation through a series of analytical and numerical simulations (COMSOL Multiphysics).

### 2.1. Tunneling Electrodes and Attractive Forces

**Figure 4:**
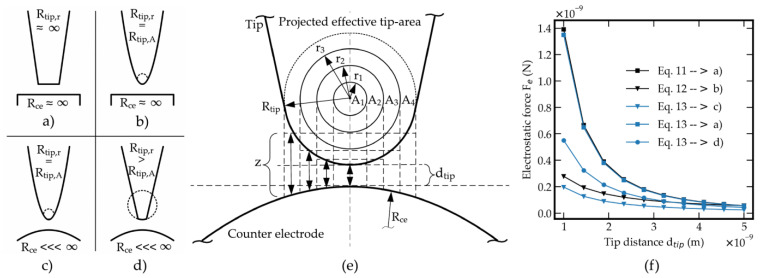
Analytical approach for the calculation of electrostatic force between tunneling electrodes. (**a**–**d**) Overview of possible tip–tip configurations. (**e**) Geometrical parameters of tip and counter electrode respectively configurations in (**c**,**d**). (**f**) Comparison of three calculation results for the plane–plane, the sphere–plane and modified equation for the sphere–sphere configuration. $R_{tip,r}$ = radius of the tip rounding, $R_{tip,A}$ = radius of the tip area, $R_{ce}$ = radius of the counter electrode, z = total effective distance of a subarea of the tip, $d_{tip}$ = the tip center distance.

The analysis of attractive forces between two tips, where at least one of the tips is freely movable, is mandatory for designing a tunneling accelerometer to prevent a snap-in at the approach of the tunneling tips. These forces act against the resetting force of the spring. The sum of the attractive forces results from the individual forces of the electrostatic force, the Van der Waals force and the capillary force. In common, all forces depend on the distance and size of the effective surfaces. This determines the height of the individual force. The electrostatic force $F_{e,pl-pl}$ between two circular capacitor plates is defined by:

(11) $$F_{e,pl-pl}=\frac{\varepsilon_{0}\;\varepsilon_{r}\;\pi \;R^{2}\;U_{t}{}^{2}}{2\;d^{2}}$$

with the radius R of the circular area, the dielectric constant $\varepsilon_{0}$, the permittivity of vacuum $\varepsilon_{r}$, the voltage $U_{t}$, and distance of the plates d. The electrostatic force between a spherical (sp) shape and plane (pl) counter plate can be calculated by (12). Since, in the case of a spherical shape, the effective surfaces do not oppose each other in a plane-parallel manner, a change in effective distance must be assumed starting from the center or the point with the shortest distance between the electrodes. For this configuration, Equation (11) cannot be applied and needs to be modified to:

(12) $$F_{e,sp-pl}=\frac{\varepsilon_{0}\;\varepsilon_{r}\;\pi \;R\;U_{t}{}^{2}}{d}$$

with the radius R of the spherical tip. The tunneling electrodes model is based on a tip-to-tip configuration. Since the preparation is carried out by the focused ion beam the resulting tips become spherical. One of the tips is made as sharp as possible with radii typically down to 10 nm. The radius of the counter tip is several 10 nm to enable a large vertical decrease of the tip shortest distance but also to provide a small attractive area. In Equation (12) only the radius of the spherical tip is taken into account. Equation (12) is, therefore, a simplified approximation for this case. To bring the calculation of the electrostatic force between a spherical tip and also a spherical counter electrode closer to the real case, the radii of both tips must be included. Moreover, it has to be distinguished between the radius of the tip rounding and the radius of the effective tip area. Figure 4 shows the illustration of these geometric parameters for the analytical approximation of different tip-to-tip configurations and in detail the real case (sphere-sphere) by considering the radii of the movable tip and the fixed counter electrode/tip.

By including both the radii of the tip rounding and the effective tip area of the two tips, the actual influence of the distance between the spherical electrode surfaces can be determined. The most significant force occurs in the shortest electrode distance area and decreases in relative terms with increasing distance from it, thus, increasing the electrode distance. The terms (11) and (12) cannot be applied for this case. Equation (13) is derived from Equation (11) and includes the rounding of the electrodes and thus the resulting relative change in distance:

(13) $$F_{e,sp-sp}=\overset{n}{\underset{i=1}{\sum }}\frac{\varepsilon_{0}\;\varepsilon_{r}\;A_{i}\;U_{t}{}^{2}}{2\;z_{i}^{2}}$$

where n is the number of subdivisions of the effective circular area of the tip and $U_{t}\;$ is the potential voltage between the electrodes. The effective subarea $A_{i}$ is given by:

(14) $$A_{i}=\left(r_{i}^{2}-r_{i-1}^{2}\right)\;\pi$$

with the radius $r_{i}$ ($r_{0}=0$, $r_{n}=R_{tip,A}$) of the subarea

(15) $$r_{i}=\left(\frac{R_{tip,A}}{n}\;i\right)$$

The effective distance $z_{i}$ per subarea is given by:

(16) $$z_{i}=d_{tip}+\Delta z_{tip,i}+\Delta z_{ce,i}$$

with the tip center distance $d_{tip}$ and the additional effective distance of the subdivision of the tip $\Delta z_{tip,i}$

(17) $$\Delta z_{tip,i}=R_{tip,r}-\sqrt{R_{tip,r}^{2}-{\left(\frac{R_{tip,A}}{n}\;\left(i-0.5\right)\right)}^{2}}$$

and the additional effective distance of the subdivision of the counter tip $\Delta z_{ce,i}$

(18) $$\Delta z_{ce,i}=R_{ce}-\sqrt{R_{ce}^{2}-{\left(\frac{R_{tip,A}}{n}\;\left(i-0.5\right)\right)}^{2}}$$

$R_{tip}$ (in the spherical case where $R_{tip,r}=R_{tip,A}$) represents the radius for the tip and $R_{ce}$ the radius of the counter electrode. In the case of $R_{tip,r}$ ≠ $R_{tip,A}$, it has to be distinguished between $R_{tip,A}$, which is the radius of the effective area and $R_{tip,r}$, which is the rounding of the tip (see Figure 4d). With Equation (13) the electrostatic force is the sum of the forces per effective subarea and its distance from the counter electrode. Figure 4 shows the comparison of the calculation approaches of the configurations plane–plane, sphere–plane, and sphere–sphere. The radii of Equations (11) and (12) have different meaning. For the plane–plane configuration, the radius refers to the tip area and for the sphere–plane model, it refers to the rounding of the sphere. Figure 4f shows the high dependence of the resulting electrostatic force and the electrodes’ parameters by including the radii of the tip rounding $R_{tip,r}$, the effective area $R_{tip,A}$, and the counter electrode $R_{ce}$. At high values of $R_{tip,r}$ and $R_{ce}$ the curve of Equation (13) approaches the result of Equation (11) of the plane-plane configuration. For values of $R_{tip,r}$ = $R_{tip,A}$ and high values of $R_{ce}$ the results of Equation (13) equal those of Equation (12) for the sphere-plane configuration. In this work, the tunneling tips can be described by spherical tips where $R_{tip,r}$ and $R_{tip,A}$ are largely equal and values of $R_{ce}$ are higher compared to $R_{tip}$ by approximately one order. The tip production with the FIB shows reproducible values of 10 nm for the movable tip and 100 nm for the counter electrode. The diagram of Figure 4f illustrates the problem of using a tip whose shape lies between a spherical and a planar profile. The application of Equation (13) shows the necessity to consider the two tips’ parameters to avoid errors regarding the electrostatic attraction. With calculation by Equation (13) any potential configuration of the tips can be chosen.

In the immediate order, the VdW forces also play a significant role in addition to the electrostatic force. The VdW forces are interactions between atoms or molecules and the resulting dipole forces at very small distances. The VdW forces for the attraction between a planar and spherical form can be calculated by the interaction energy between the tips as a function of the distance d [55]:

(19) $$U_{VdW}\left(d_{tip}\right)=-\frac{H}{6}\;[ln\left(\frac{{\left(d_{tip}+R_{2}+R_{1}\right)}^{2}-{\left(R_{2}+R_{1}\right)}^{2}}{{\left(d_{tip}+R_{2}+R_{1}\right)}^{2}-{\left(R_{1}-R_{2}\right)}^{2}}\right)\;+\frac{2\;R_{1}\;R_{2}}{{\left(d_{tip}+R_{2}+R_{1}\right)}^{2}-{\left(R_{2}+R_{1}\right)}^{2}}\;+\frac{2\;R_{1}\;R_{2}}{{\left(d_{tip}+R_{2}+R_{1}\right)}^{2}-{\left(R_{1}-R_{2}\right)}^{2}}]$$

with H as the Hamaker constant, $R_{1}$ and $R_{2}\;\left(\mathrm{according}\;\mathrm{to}\;R_{tip}\right)$ the constant radii of the tips. The VdW force is the negative of the derivative of the potential energy function and is therefore defined by

(20) $$F_{VdW}\left(d_{tip}\right)=\frac{32\;H\;R_{1}^{3}\;R_{2}^{3}\;\left(d_{tip}+R_{2}+R_{1}\right)}{3\;d^{2}\;{\left(d_{tip}+2\;R_{2}+2\;R_{1}\right)}^{2}\;{\left(d_{tip}{}^{2}+\left(2\;R_{2}+2\;R_{1}\right)\;d_{tip}+4\;R_{1}\;R_{2}\right)}^{2}}$$

VdW forces are limited to distances of a few hundred Å. The radii refer to the rounding of the tip in the sense of a spherical shape. The radius includes the effective area or the increasing distance of the surface units starting from the shortest distance. Equations (19) and (20) require the radii of both tips to be facing to each other.

A further force only plays a role under atmospheric conditions, as it includes the capillary effect due to the surrounding gaseous or liquid medium. The resulting capillary force is based on the molecular forces at the interfaces of the substances and can be defined by [56]

(21) $$F_{cap}=\frac{4\;\pi \;\gamma_{liquid}\;R_{tip}}{1+\left(d_{tip}/h\right)}$$

where $\gamma_{liquid}$ is the interfacial energy of the transition and h is the thickness of the layer of surrounding medium between the surfaces. When applied in air and with regard to the resulting water film due to the humidity, the capillary force takes the dominant role compared to the electrostatic and VdW forces. In vacuum, the electrostatic and VdW forces have a dominant influence over the capillary force. Thus, this force is not considered within this work since all experiments were performed under vacuum conditions to protect the tunneling tips. Table 2 shows the parameters of the tips for calculation of the attractive forces.

**Figure 5:**
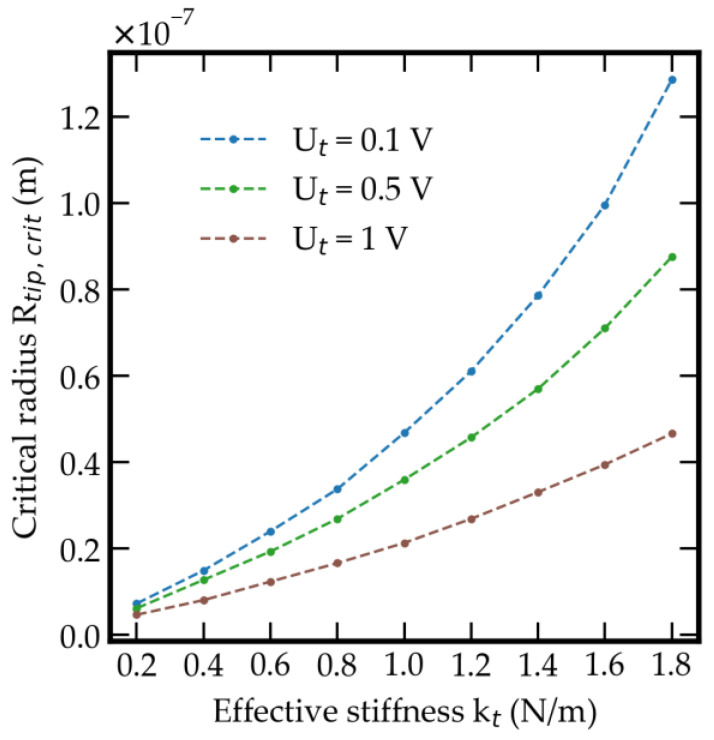
Effective stiffness $k_{t}$ vs. critical radius of $R_{tip,crit}$ at different tunneling bias voltage $U_{t}$ to reach an effective tunneling distance of 10 Å.

Obviously, the effective area for attractive forces is an essential parameter for investigating the approach of the tunneling electrodes. To avoid a snap-in effect, the sensor system must be designed with a minimum effective stiffness $k_{t}$. The effective stiffness $k_{t}$ is defined by the deflection of the tunneling tips through the impact of the attractive forces and has to be high enough to prevent a snap-in effect at the approach of the tunneling electrodes down to a final distance of several Å. Thus, $k_{t}$ is one of the most significant values for the design of the sensor. Since there are no fixed electrodes like in a scanning tunneling microscope, it is important to distinguish between a setpoint and a snap-in point. The snap-in point is defined by the distance, where the restoring spring force is not able to withstand the increasing attractive forces. More precisely, there is no longer an intersection between the course of the opposite forces as a function of the distance. The setpoint defines the actual distance between the tips due to the equilibrium between restoring and attractive forces. For practical purposes, the setpoint is always lower than the actuator point, which refers to the distance of the tips changed by the movement of the actuator and is independent of the attractive forces. If the setpoint reaches the snap-in point the system is no longer able to prevent a snap-in and the tip is locked by the attractive forces. Figure 5 shows the radius $R_{tip,crit}$ vs. the effective stiffness $k_{t}$ to prevent a snap-in at a tunneling distance of 10 Å for different levels of tunneling bias voltage $U_{t}$ based on Equations (13) and (20), confirming the mandatory requirement for a low tip radius $R_{tip}$. The effective stiffness $k_{t}$ leads to an essential parameter of the spring-mass system. Because of the dependence of $k_{t}$ and the sensitivity of the spring-mass system for applied accelerations, it has to be considered that a low spring stiffness is chosen. The tunneling effect takes place at a tip distance in a range of several Å. To ensure a tunneling bias voltage up to 1V, all pairs of radius and stiffness below the 1V-line are possible to reach an effective tunneling distance of 10 Å. Based on these limits, exemplarily for a stiffness of 1 N/m at $U_{t}$ = 1 V, a maximum radius of 20 nm is allowed. This enables a tip movement up to a final distance of 10 Å, where the tunneling effect has a high sensitivity due to its exponential correlation between the tunneling current and the tip distance. To summarize, the smaller the radius and the higher the stiffness, the smaller is the possible distance and the higher the measuring range related to an applied acceleration.

### 2.2. Mechanics

**Figure 6:**
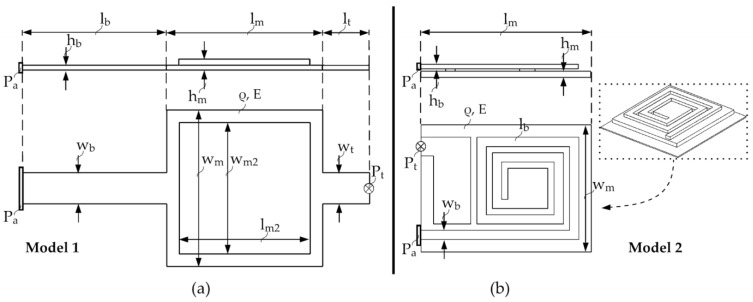
Two concepts of the sensor structure with parameters $l_{b}$ beam length, $h_{b}$ beam thickness, $w_{b}$ beam width, $l_{m}$ mass length, $h_{m}$ mass thickness, $w_{m}$ mass width, ρ material density, $l_{t}$ tunneling beam length, $w_{t}$ tunneling beam width, $P_{a}$ system anchor point, and $P_{t}$ point of tunneling electrodes. (**a**) Single beam model. (**b**) Spiral beam model.

To operate the tunneling effect in reliable conditions, a symmetrical sensor structure with a single beam (Figure 6a) and high lateral stability is chosen. This work aims to reach a high grade of miniaturization. For this, an optimized sensor structure with an area requirement close to 50 × 50 µm^2^ with a spiral beam concept (Figure 6b) should be considered. This enables the creation of a long beam and a smaller mass with a smaller overall area requirement. Both concepts are shown in Figure 6. They are designed in a way that the primary displacement operates in just one direction. Since the sensitivity of the tunneling part is very high and movements of just some Å are needed, a maximum of simplification related to the spring-mass system is aimed for. Obviously, there is a lower lateral stability in model 2 (Figure 6b). To determine the sensor structure’s geometrical parameters, three different stiffness constants have to be considered. The already shown $k_{t}$ depends on the attractive force of the tunneling tip at distances of a few nm, $k_{a}$ depends on the seismic mass and deflection of the tunneling tip by an applied acceleration, and $k_{e}$ is based on the deflection of the actuator by the electrostatic force.

The operation depends mainly on the stiffness of the system in terms of the deflection and attractive forces of the tunneling electrodes. Starting from an already determined minimum value for $k_{t}$, the spring-mass system parameters and, in particular, the stiffness $k_{a}$ can be determined. If the restoring force is too low, a snap-in occurs before a few nm distance is reached. On the one hand, it must be considered that the value for the stiffness $k_{t}\;$ does not fall below a minimum value, and on the other hand, $k_{a}$ has to be determined in a way that the measuring range and the sensor’s sensitivity are as high as possible. The measuring range and the sensitivity depend significantly on the seismic mass, the spring stiffness, and the maximum measuring range and can be calculated with the analytical formulae for model 1 (Figure 6a). The calculation for $k_{t}\;$ and $k_{a}$ results from the spring parameters, the resulting momentum of the mass, and the position of the tunneling electrode in the system. The deflection of the tunneling electrodes $x_{tip}$ is a superposition of the beam deflection and the spring’s inclination together with the distance to the end of the beam.

(22) $$x_{tip}=x_{b}+x_{t}$$

with $x_{b}$ as the deflection of the spring:

(23) $$x_{b}=\frac{M\;l_{b}^{2}}{2\;E\;I}$$

with $l_{b}$ as the length of the beam, E the Young’s modulus, I the area moment of inertia, and the exerting moment M (for $M_{a}$ or $M_{t}$). $M_{a}$ refers to the force $F_{a}$ caused by an applied acceleration a to the seismic mass m and $\;M_{t}$ to the attractive force $F_{t}$ between the tunneling electrodes:

(24) $$M_{a}=F_{a}\;\left(\frac{l_{m}}{2}+l_{b}\right)$$

(25) $$M_{t}=F_{t}\;\left(l_{m}+l_{t}+l_{b}\right)$$

with $l_{m}$ as the length of the mass and $l_{t\;}$ the lenght of the additional short beam for the tunneling electrodes. $x_{t}$ is the additional deflection through the spring’s inclination and distance between the position of the tunneling electrodes and the end of the beam:

(26) $$x_{t}=\left(l_{m}+l_{t}\right)\;sin\left(\varphi \right)$$

with the inclination sin(φ) = φ for small angles at the end of the spring:

(27) $$\varphi =\frac{M\;l_{b}}{E\;I}$$

with $M_{a}$ or $M_{t}$ for M. The contributions to the total deflection at the location of the tunneling section result in case of an applied acceleration through

(28) $$F_{a}=m\;a$$

with the acceleration *a* and the mass *m*

(29) $$m=l_{m}\;w_{m}\;h_{m}\;\rho$$

from the width $w_{m}\;$ and thickness $h_{m}$ of the seismic mass and ρ the density of the material. Besides, the deflection depends on the area moment of inertia I of the spring:

(30) $$I=\frac{w_{b}\;h_{b}{}^{3}}{12}$$

with $w_{b}$ and $h_{b}$ for the width and the height of the beam. The equivalent spring stiffness k for the system is given by

(31) $$k=\frac{F}{x_{tip}}$$

$x_{tip}$ refers to the total deflection of the tunneling section. The force F refers to the seismic mass m and the applied acceleration a  or the sum of the attractive forces. From the already determined value for $k_{t}$, as the stiffness related to the attractive forces of the tunneling electrodes, the geometric quantities of the spring-mass system can be derived. Through Equation (31) the relation for $k_{t}$ and $k_{a}$ as a function of the different lengths can be determined:

(32) $$k_{t}=\frac{2\;E\;I}{l_{b}\;\left(l_{b}+l_{m}+l_{t}\right)\;\left(l_{b}+2\;\left(l_{m}+l_{t}\right)\right)}$$

(33) $$k_{a}=\frac{2\;E\;I}{l_{b}\;\left(\frac{l_{m}}{2}+l_{b}\right)\;\left(l_{b}+2\;\left(l_{m}+l_{t}\right)\right)}$$

To prevent a snap-in at the tunneling approach until 10 Å, a minimum value of $k_{t}$ determines the lengths $l_{b}$ and $l_{m}$ and also the resulting stiffness $k_{a}$. Table 3 shows the given, chosen, and calculated values of the mechanical sensor system and the comparison of analytical and numerical results for the tunneling tip’s desired deflection $x_{tip}$ in the range of 1 Å at 1 g of the applied acceleration.

The relationship between the deflection x(t) and the applied force F(t) of the sensor system can be represented as the following differential equation as a function of time *t*:

(34) $$F\left(t\right)=m\;\frac{d^{2}x}{dt^{2}}+c\;\frac{dx}{dt}+k\;x\left(t\right)$$

For the characterization of the sensor, periodic forces can be introduced by either a shaker, static forces by electrostatic self-test actions, or constant forces by the gravitational field. The mass, the damper, and the spring of the system affect the course of the deflection. Some mechanisms such as thermoelastic damping, intrinsic friction effects, or the squeeze-film theory are worthy of consideration to determine the damping constant. In particular, for squeeze-film damping, some publications with relation to tunneling sensors are available [36,57,58]. Nevertheless, there is no evidence for the relationship between deflection and squeeze damping factor. Since the movement takes place far below the mean free path of a particle in air at atmospheric pressure ($\lambda_{air}$ = 68 nm), and measurements are performed in vacuum conditions, the influence of a squeeze-film effect can be neglected. The resulting losses are minor and will be neglected initially for the design of the sensor. For the estimation of the resolution limit, the calculation of the thermal noise equivalent acceleration (TNEA) is used and defined by:

(35) $$TNEA=\sqrt{\frac{4\;K_{b}\;T\;\omega_{0}}{m\;Q}}$$

with $K_{b}$ as the Boltzmann constant, T the temperature, $\omega_{0}$ the angular frequency, m the seismic mass, and Q the quality factor of the system. Since the damping constant is assumed to be very low, the quality factor is high and chosen as a moderate value of 50.

### 2.3. Electrostatics

**Figure 7:**
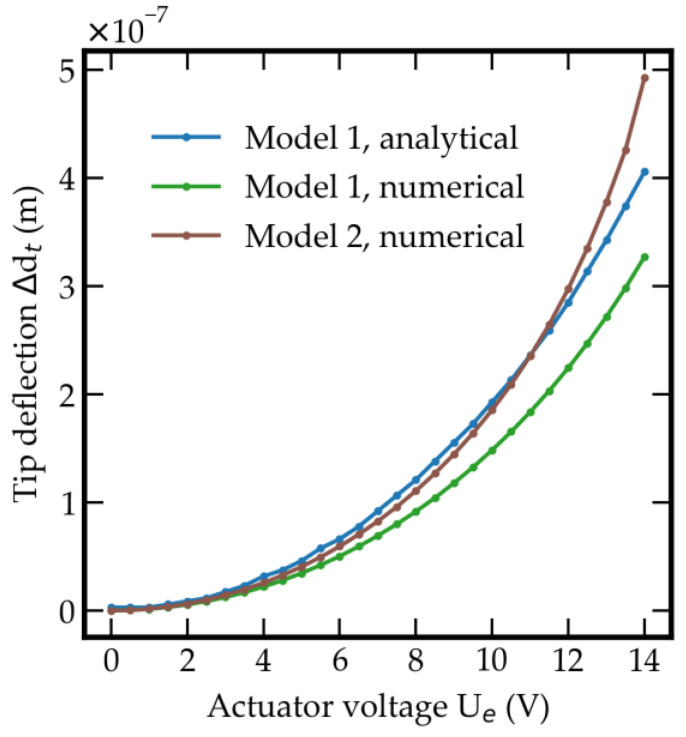
Comparison of tip deflection by electrostatic actuator for models 1 and 2.

In addition to Equation (11) the electrostatic force $F_{e}$ exerted by the actuator can be determined by:

(36) $$F_{e}=-\frac{1}{2}\;\varepsilon_{0}\;\varepsilon_{r}\;\frac{A_{e}}{d_{e}^{2}}\;U_{e}^{2}$$

with a rectangular or quadratic area of the capacitor $A_{e}$, the dielectric constant $\varepsilon_{0}$, the permittivity of vacuum $\varepsilon_{r}$, actuator voltage $U_{e}$, and distance of the plates $d_{e}$. Two counter plates are placed below the mass and the beam for primary and self-test electrostatic actions. The primary actuator leads the sensor into operational conditions and keeps the voltage constant in the static mode. The tunneling section’s fabrication tolerances lead to a starting distance of the tunneling electrodes between 30 and 300 nm. Moreover, depending on the system stiffness, the required voltage amounts to a range between 4 and 13.5 V. Due to its preload, this actuator can also be used as a control actuator. The self-test actuator can be used for simulating a disturbance by voltage or electrostatic force. Table 4 shows the parameters of the electrostatic actuator and in particular the result for the necessary actuator voltage, depending on the initial tip distance. The resulting course of the tunneling electrode gap as a function of the actuator voltage is shown in Figure 7.

## 3. Fabrication

The fabrication of the sensor is carried out by the foundry service PolyMUMPs from MEMSCAP Inc. and subsequent micro structuring of the tunneling electrodes with a focused ion beam. PolyMUMPs is a surface micro-machining process with three polysilicon (PolySi) layers, two sacrificial layers, and a metal layer. The minimum structure size for PolyMUMPs is 2 µm. The standardized process procedure with strict adherence to the design rules ensures low manufacturing times and costs. The FEI Helios Nanolab 600 dual beam, consisting of an electron and ion beam column ($Ga^{+}$) with an additional gas injection system and a metal–organic precursor ($MeCpPtMe_{3}$), is used for post-processing and integration of the tunneling tips.

### 3.1. Sensor Structure

**Figure 8:**
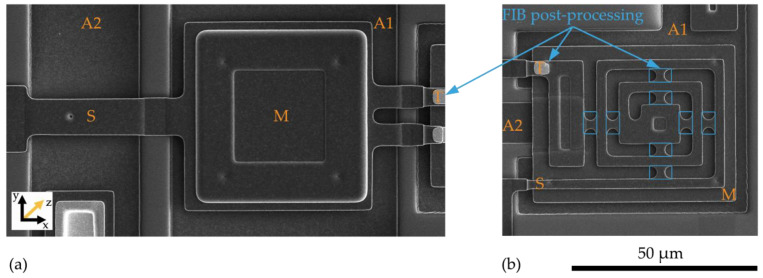
PolySi Sensor structures with (A) electrostatic actuators, (M) seismic mass, (S) spring, (T) position of tunneling electrodes. (**a**) Single beam sensor structure. (**b**) Spiral beam sensor structure.

The sensor structures are placed on a 5 × 5 mm^2^ die, bonded subsequently by a Wire Bonder G5 Single (F&K Delvotec) to a breakout printed circuit board (PCB) for electrical connectivity during FIB processes and subsequently operational testing. Figure 8 shows the two polysilicon sensor structures fabricated by the PolyMUMPs process according to the geometric values of Table 3. The spring (S), seismic mass (M), actuator counter pads (A), and position of the tunneling section (T) are marked. The FIB technology offers flexible micro structuring of the SMM layers so that the spring can be shortened or thinned out and the seismic mass can be adjusted in size. At the marked FIB sections in Figure 8b, the structure was subsequently released. The connections between the beam sections were provided to prevent sticking to the structure during etching of the sacrificial layer, transport and mounting the MEMS to the PCB. Additionally, adjustments to the stiffness of the mechanical system can be realized. The integration of the tunneling electrodes is performed at the signed positions (T) by 3D nano patterning with the FIB.

### 3.2. Tunneling Electrodes

Several different variants were tested for the production of the tunneling electrodes. These mainly relate to using the platinum organic precursor material ($MeCpPtMe_{3}$) of the gas injection system. An additional variant was investigated by structuring the gold pads applied by the SMM process. In the following, the fabrication method is described in detail (Table 5). For deposition and patterning processes with the $Ga^{+}$ ion column, an acceleration voltage of 30 kV was used. Depending on the process step, the ion current was varied at 1.5–260 pA. The effective radius of the final tunneling tip of step 5 is about 10 nm, with a distance to the lower counter electrode of about 200 nm. The final radii of the electrodes have tolerances of 10 ± 5 nm. Initial distances range from 30 nm to 300 nm. Depending on the spring stiffness and size of the electrostatic actuator, voltages in the range of 4 V to 13.5 V are necessary to overcome the initial spacing and guide the electrodes to a tunneling distance of a few nm.

## 4. Experimental Procedure

**Figure 9:**
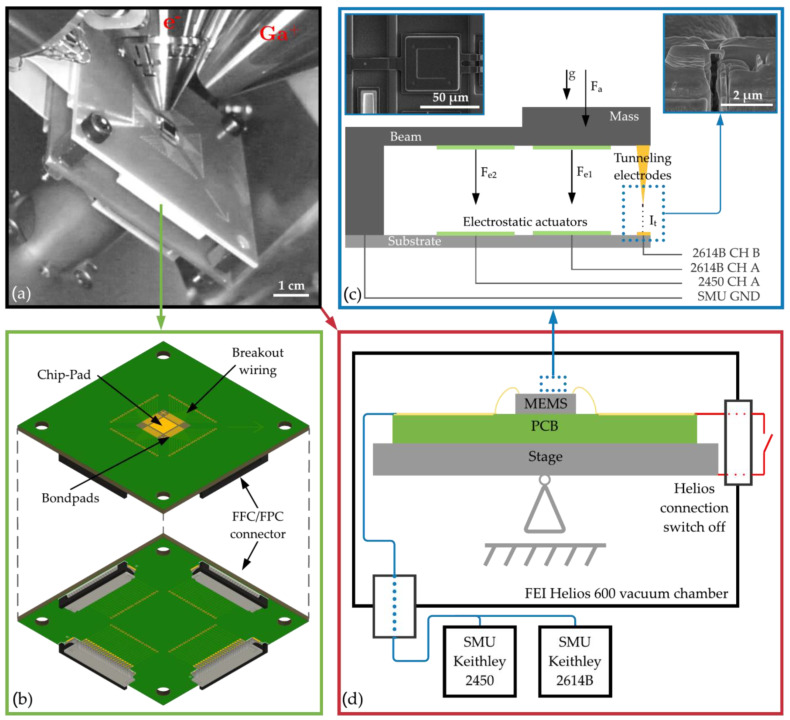
Test setup in the Helios vacuum chamber (**a**) and in detail the circuit board (**b**), the wiring of the sensor structure, and the FIB tunneling tips (**c**), and the schematic of the test setup (**d**).

An overview of the test setup for connecting the sensor structure to the measuring equipment is shown in Figure 9. The circuit board with the bonded chip is mounted in the vacuum chamber of the Helios (Figure 9a). After processing the sensor structure and fabricating the tunneling electrodes with the FIB, the PCB is completely separated from the electronics of the Helios (Figure 9d). The switch outside the vacuum chamber is connected to the stage and the PCB via the cable gland. As shown in Figure 9b, the PCB has FFC/FPC (flexible flat cable/ flexible printed circuit) connectors (bottom) whose pins are connected to the bond pads of the chips via the breakout trace (top). Figure 9d illustrates the schematic of the measurement system. The measurements are carried out with the high-precision SMU Keithley 2614B and Keithley 2450 from Tektronix. The two-channel 2614B is connected to the primary actuator and the tunneling counter electrode, and the 2450 to the second actuator for self-test actions (Figure 9c). During fabrication of the tunneling electrode, all cables are short-circuited to the stage GND (Ground). For the measurement, the GND is linked to the spring-mass system. The connection from the SMU to the MEMS chip is made via a cable lead-through on the vacuum chamber. The sensor chip is wired using Teflon cables to minimize outgassing and contamination during the processing of the sensor structure inside the vacuum chamber. All measurements are performed using different Lua scripts that automate the execution of a loop on the SMUs. The different stages (reaching the operating point, reaching a current threshold, reaching a specified number of measurement points, sweeping the actuator voltage for self-test actions) of the measurements are run through by querying the measured values. All measurements are carried out under high vacuum conditions to prevent contamination of the tunneling tips and keep the environmental conditions as constant as possible. For the sensor-technical implementation of the susceptible tunneling effect, sources of disturbances must be avoided as far as possible.

## 5. Results and Discussion

**Figure 10:**
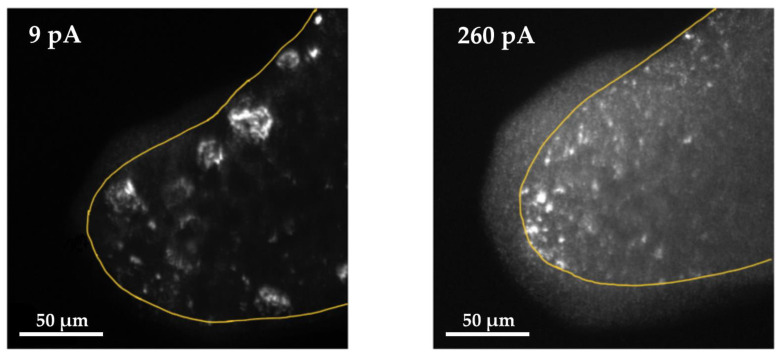
TEM analysis of two FIB-PtC tips shows the difference in distribution of carbon (dark) and platinum (bright) in the material structure depending on the chosen ion current. Left: 9 pA ion current. Right: 260 pA ion current. The accumulation outside the edge of the tip is caused by the deposition of C-H during the TEM analysis.

All of the following measurements show raw current signals, directly measured without amplification or conversion into voltage signals. It can be seen that both negative and positive tunneling bias voltages were used to show reproducibility and independency of current direction due to the use of the same material on both electrodes. This may be an essential factor for the ongoing development of the circuitry of the sensor. In addition, measurements show leakage currents in the range of up to |9 pA| due to supply lines, actuation voltage, damage of the substrate by the ion beam (implantation of $Ga^{+}$), and offsets by the SMUs. Reliable operation of the tunneling section is possible up to tunneling currents of approximately 150 pA due to the high resistance of the metal–organic electrodes depending on the tunneling bias voltage of 1 V. Besides the fabrication of ultra-thin tips, carbon contamination is the most challenging difficulty for the realization of the tunneling effect with metal–organic materials. It is known that the deposited material from a metal–organic precursor is highly contaminated by $sp_{2}$/$sp_{3}$-carbon structures [54]. For reliable tunneling, platinum atoms need to be located as close as possible to the edge of the tip. Figure 10 shows the darkfield images of the TEM (transmission electron microscope) analysis and the difference in distribution of the carbon (dark) and platinum (bright) elements in the material structure depending on the chosen ion current. Higher currents lead to much higher homogeneous distribution and lower currents to the growth of large platinum grains. Details will be investigated in another survey. 

**Figure 11:**
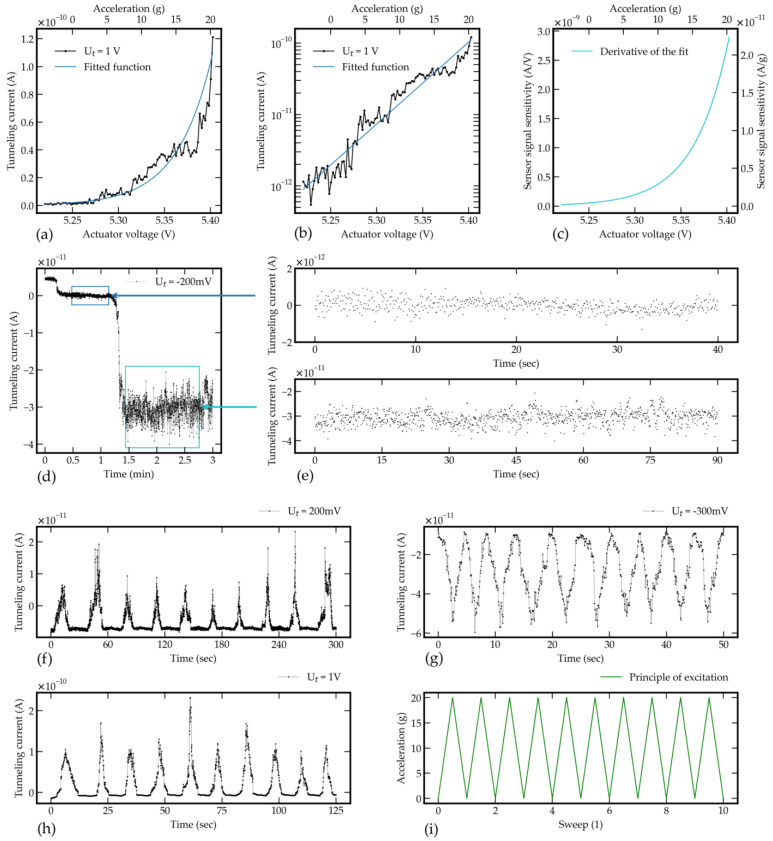
Measurements of tunneling current. (**a**,**b**) The tunneling effect verification on the sensor structure. (**c**) The fitted function of the sensor sensitivity. (**d**) Behavior under static load of an equivalent acceleration of 20 g. (**e**) Difference in signal noise depending on tunneling distance. (**f**–**h**) Response of the tunneling sensor by ramp excitation by electrostatic self-test action for different tunneling bias voltage and initial tunneling tip distances. (**i**) Principle of multiple excitation.

Figure 11 shows the tunneling effect verification on the sensor structure (a,b), the fitted function of the sensor sensitivity (c), behavior under static load of an equivalent of 20 g (d), the difference in signal noise depending on tunneling distance (e), response of the tunneling sensor by ramp excitation by electrostatic self-test action for different tunneling bias voltage and initial tunneling tip distances (f–h). In Figure 11a, the tunneling bias voltage is set to 1 V. To derive the exponential fitted function, the measured values from Figure 11a are shifted upwards by 10 pA to compensate for the offset or minimum measured value in the negative region. According to phase 1 in Figure 3, the voltage of the electrostatic actuator is increased in 100 mV steps until the initial distance between the tunneling electrodes is largely overcome at 5.20 V. The last nm, until the tunneling effect occurs (at approximately 5.25–5.27 V) and subsequently a tunneling current of 110 pA is reached (at 5.40 V), is overcome with a more sensitive step size of a few mV. Figure 11a shows the measuring range of 20 g. The acceleration equivalent to the actuator voltage can be determined from the calculations for the deflection of the sensor structure (deflection/g) and the electrostatics (deflection/V). According to Figure 11h, the maximum tunneling currents at a tunneling bias voltage of 1 V are in the range of 100–200 pA. The differences in the peaks show a clear dependence on the increased sensitivity of the tunneling effect by reducing the tunneling distance. Other measurements show that a continued reduction of the tunneling distance, measured by a further increase of the tunneling current, leads to a snap-in of the electrodes. The measuring range can theoretically be increased to the snap-in equivalent tunneling current but is limited to a moderate value of 20 g. Thus, the measuring range of a tunneling accelerometer in terms of the displacement is limited to the maximum tunneling distance and the snap-in point. Previous works used a much larger sensor area with long beams and a large proof mass to measure very small accelerations at high sensor signal sensitivity. Therefore, the opposing measuring range in these works is much lower with a value of 10 mg at Baski et al. [28], and Zavracky et al. [41], 1 mg at Liu et al. [42,43], 600 µg at Strobelt [45], and 10 µg to 10 mg at Patra et al. [36]. In the publications of Kenny et al. [25,26] and Rockstad et al. [33] no data on the measuring ranges are available, but it can be assumed that these are in a similar range. Compared to the other works on tunneling accelerometers, it tends to be observed that all these works present the highest values for the resolution up to 10 ng/$\sqrt{Hz}$ [25] due to a large seismic mass and lower thermal noise amplitude. Comparable measuring ranges to the present work are provided by Yeh et al. [30,31,32] with −20–10 g, Burgner et al. [39] and Miao et al. [47] with ±10 g, and Dong et al. [46] with 1 g. These works show a small sensor core area, resulting in higher stiffness and lower sensitivity. Furthermore, Kubena et al. [34,35] indicate a much higher measuring range of 10^4^ g due to an increased stiffness and without an additional proof mass. Figure 11b shows the proof of the exponential dependence of the tunneling effect based on the logarithmic scale and the fitted line function. The sensitivity of the sensor signal can be determined by the derivative of the fitted function and is shown in Figure 11c. Due to the exponential dependence of the tunneling effect, the sensitivity also depends exponentially on the electrode distance. At an actuator voltage of 5.25 V (sensor operating point) and a corresponding acceleration of 0 g, a very low sensitivity of 51.8 pA/V or 0.4 pA/g is given. As the electrodes are further approached, the sensitivity increases to 374.5 pA/V or 2.9 pA/g at 5.325 V/10 g and increases significantly to a value of 2.71 nA/V or 20.9 pA/g at 5.4 V/20 g. Comparison of the sensitivity data to all previous works is difficult due to very different representations of these results. However, Dong et al. [46] show a slope of the tunneling current from 0.9 nA at 0 g (sensor operating point) to 1.7 nA at 1 g. Compared to the present work, the signal sensitivity is two orders of magnitude higher approximately due to a larger proof mass. Additionally, the use of a metallic electrode allows improved exploitation of the tunneling current range. A much higher sensitivity of 30 µA/g up to 300 µA/g is given by Kenny et al. [25,26] due to a significant larger sensor size. Based on the shown values, it can be seen that the performance data of the sensor depends on the sensor size or stiffness and the resulting deflection due to an acting acceleration limited by the maximum tunneling distance of the electrodes.

According to phase 2 in Figure 3, the position of the tunneling electrodes and the tunneling current for the operation of the sensor must be kept constant. Figure 11d shows the static characteristics of the sensor structure at the operating point and the increase of the tunneling current of about 30 pA due to an additional load of several g caused by the electrostatic actuator at a tunneling voltage of −200 mV. Figure 11e shows the two levels of the measurement in detail. At the offset current equivalent setpoint of 5.03 pA_rms_, the peak-to-peak noise amplitude is 2.22 pA_p-p_ with a standard deviation of 333 fA. The course of the tunneling current after the step due to an additional load shows the increasing sensitivity with approach of the electrodes. The tunneling distance reduction to a tunneling current of 35.8 pA_rms_ results in a peak-to-peak noise amplitude of 19.5 pA_p-p_ and a standard deviation of 2.83 pA. According to this measurement, an acceleration-equivalent static noise amplitude can be derived. A clear increase of the noise amplitude with a reduction of the tunneling distance is recognizable due to an increasing tunneling effect sensitivity.

Due to the high compression of a possible tunneling current range down to several 10 pA by using metal–organic electrodes, in particular, noise sources that are independent of the movements overlay the measurement signal with a significant impact. This leads to a strong decrease in the sensor signal resolution and shows the clear requirement for metallic electrodes with low inherent resistance. The comparison with previous work on tunneling accelerometers (Section 1.2) proves a high resolution [25,26,33,42,43,44,45,47] due to the high sensitivity of the tunneling effect, which is therefore not fully exploited in the present work.

Furthermore, the sensor structure is loaded several times by the electrostatic actuator with an equivalent acceleration. This is done by increasing and decreasing the voltage of the actuator several times according to the principle of excitation shown in Figure 11i. In this sense, the reproducibility of the sensor signal can be demonstrated based on the course of the tunneling current. The measurement is based on different tunneling voltages and voltage steps at the actuator. Therefore, Figure 11f–h show, on the one hand, the electrostatic load on the sensor structure with an equivalent acceleration of 20 g, and on the other hand the dependency of the tunneling currents’ amplitude on the applied tunneling bias voltage, according to Equation (10). As shown in Section 4, the SMUs are controlled automatically by the corresponding measurement algorithms. The time required per loop is the same for all measurements. The different total time requirement per measurement in Figure 11f–h can therefore be explained by the choice of different magnitudes of voltage steps on the actuator. The smaller the voltage step, the greater the time requirement. With decreasing step size, the resolution of the measurement increases due to an overall larger number of measuring points. The slightly different course of the tunneling currents is due to a varying initial distance of the tunneling electrodes. In Figure 11f,h the deflection leads to dipping in the tip into the tunneling distance. In Figure 11g the electrode oscillates and stays within a distance where the tunneling effect occurs. 

With respect to previous work, the results show that instead of increasing the sensor resolution of an accelerometer [25,26,33,42,43,44,45,47], the highly sensitive tunneling effect can also be used to miniaturize the required sensor area. The sensor area in the present work is much smaller than in previous works like shown in Table 1. With regard to the work of Kubena et al. [34,35] with a slightly larger size of the sensor core area than in the present work, further miniaturization was also achieved, with lower system stiffness due to much smaller radii of the electrode tips. However, the measurements show lower resolution due to the use of metal–organic materials with high inherent resistance. This confirms the need for metallic electrodes for tunneling application when using low tunneling bias voltages and moveable parts. To summarize, the tunneling effect can be demonstrated on the miniaturized tunneling sensor structure by different types of measurements. The acceleration-sensitive characteristic of the spring-mass system can be shown by static loading and keeping a certain tunneling distance. The multiple loading of the system at different tunneling voltages leads to the expected differences in the amplitude of the sensor signal, according to Equation (10). The course of the tunneling current shows reproducible values regarding to an acceleration-equivalent loading of the spring-mass system. Since no snap-in effect occurs at the approach of the tunneling electrodes, the effective system stiffness is chosen in an appropriate range. The reduction of the tunneling distance leads to a higher sensitivity due to the exponential dependence of the tunneling effect.

## 6. Conclusions

The further miniaturization of acceleration sensors reaches its limits using conventional transducer principles due to scaling laws when the sensor structures are isometrically reduced in size. In this work, the miniaturization potential of acceleration sensors was investigated using the highly sensitive tunneling effect. The theoretical design of the sensor structures requires an in-depth analysis of the relationships between tunneling effect, geometric shape, and size of the spring-mass structures, and the electrostatic actuator parameters. A crucial aspect concerning the tunneling distance are the attractive forces between the tunneling electrodes as a function of the electrodes’ geometric shape. The tunneling electrodes are implemented after fabrication of the sensor structures using MEMSCAP Inc. foundry service PolyMUMPs by deposition of metal–organic precursor material ($MeCpPtMe_{3}$) using a focused ion beam ($Ga^{+}$) with a gas injection system. This led to significant challenges generating electrode tips of few nm in radius. An analysis of the metal–organic microstructure and suitable parameters of the FIB is required to ensure the suitability of the electrodes for the tunneling effect. This showed the necessity of high ion currents (260 pA, 30 kV) for an increased platinum content in the microstructure and a homogeneous distribution of the platinum grains. Using the metal–organic electrodes, tunneling currents up to 150 pA were measured reliably, depending on the tunneling voltage. The limitation is due to the electrodes’ high material resistance, especially because of the tip radii of a few nm. The research results follow the essential requirement for metallic “pure” materials since the metal–organic materials significantly limit the measuring range. In comparison, a scanning tunneling microscope with a metal tip achieves tunneling currents up to some 10 nA at a low tunneling bias voltage of 100 mV.

Electrode tips with radii down to 10 ± 5 nm were fabricated, and initial electrode spacings of about 30 nm to 300 nm were achieved. This corresponds to operating voltages in the range of 4 to 13.5 V, depending on the electrostatic actuators’ size.

To prevent contamination of the tunneling tips and to ensure constant ambient conditions, measurements were performed in vacuum environment. For operation in atmospheric conditions, the sensor structures need to be encapsulated to prevent contamination of the electrode tips.

Following the objective of the work, the tunneling effect was demonstrated at highly miniaturized structure sizes of several 10 × 10 µm^2^. The stiffness of the structures is limited by the attractive forces on the tunneling electrodes. Tunneling bias voltages from 200 mV to 1 V were applied to the sensor and a measuring range of 20 g was realized. The metrological characterization showed the exponential dependence between tunneling current and electrode distance. Thus, the sensor signal sensitivity ranges from 0.4 pA/g at 0 g in the sensor operational point up to 20.9 pA/g at 20 g. Due to the increasing sensitivity of the tunneling effect, the signal noise increases significantly with the shortening of the tunneling distance. Using self-test actions, the sensor structures were loaded or excited with an equivalent acceleration, and in this way, the acceleration-sensitive function was demonstrated. The multiple excitation at different tunneling bias voltages shows a varying tunneling current amplitude, which is in good agreement with the calculations. The findings by the investigation in this work show the high potential for miniaturization of accelerometers using the tunneling effect compared to current sensors based on other transducer principles.

## Figures and Tables

**Table 1:** Comparison of parameters of already published works on tunneling accelerometers.

**Author**	$\mathrm{Area}\;[mm^{2}]$	Range [g]	$\mathrm{Resolution}\;[g/\;\sqrt{Hz}]$	Technology	Year
Baski et al. [28]	1600	10^−2^	10^−4^	Test setup	1988
Waltman et al. [29]	1800	1	10^−5^	Test setup	1989
Kenny et al. [25,26]	ca. 224	n/a	10^−8^ (1 kHz)	BMM	1990–1991
Yeh et al. [30,31,32]	0.16	−20–10	0.25 × 10^−3^ (2 kHz)	BMM	1995–1998
Rockstad et al. [33]	ca. 168	n/a	10^−8^ (100 Hz)	BMM	1996
Zavracky et al. [41]	100	10^−2^	n/a	BMM	1996
Kubena et al. [34,35]	0.0033	10^4^	8.3 × 10^−4^ (500 Hz)	SMM	1996–1999
Liu et al. [42,43]	ca. 52	10^−3^	20 × 10^−9^ (1.5 kHz)	BMM	1998–2001
Hartwell et al. [44]	1.5	n/a	20 × 10^−6^ (100 Hz)	BMM	1998
Strobelt [45]	ca. 36	6 · 10^−4^	2.5 × 10^−6^	BMM	2000
Burgner et al. [39]	ca. 1	±10	n/a	SOI	2005–2009
Dong et al. [46]	1.21	1	5 × 10^−4^ (1.25–100 Hz)	BMM	2005
Miao et al. [47]	1.21	±10	15 × 10^−6^	BMM	2007
Patra et al. [36,37]	0.36–0.96	10^−5^–10^−2^ (calc.)	3.61–9.84 × 10^−6^ (calc.)	SMM	2009
Patra et al. [38]	ca. 0.04	0.027–0.343	2.97 × 10^−6^ (calc.)	SMM	2010
This work	0.0023–0.003	20	2.4–3.4 × 10^−3^ (calc.)	SMM	2021

**Table 2:** Parameters of the tunneling tips for calculation of attractive forces.

Parameters	Variable	Results
Radius of the tip [nm]	$R_{tip}$	2–130
Radius of the counter tip [nm]	$R_{ce}$	≈100
Tip distance [Å]	$d_{tip}$	5–30
Tunneling bias voltage [V]	$U_{t}$	0.1, 0.5, 1
Vacuum permittivity [Vm/As]	$\varepsilon_{0}$	8.8541878128 × 10^−12^
Relative permittivity	$\varepsilon_{r}$	1
Hamaker constant [J]	H	10^−19^

**Table 3:** Mechanical parameters of the sensor concepts.

Parameter	Variable	Model 1		Model 2
Beam length [μm]	$l_{b}$ $/\;l_{t}$	46/15		218
Beam width [μm]	$w_{b}$ $/\;w_{t}$	10		3
Beam thickness [μm]	$h_{b}$	1.5		1.5
Mass length [μm]	$l_{m}/\;l_{m2}$	50/42		55
Mass width [μm]	$w_{m}/\;w_{m2}$	50/42		41
Mass thickness [μm]	$h_{m}$	3.5		2
Mass [kg]	m	1.7 × 10^−11^		1.05 × 10^−11^
Quality factor [1]	Q	≈50		≈50
Young’s modulus [GPa]	E	158		158
Material density [kg/m^3^]	ρ	2330		2330
Results		analytical	numerical	numerical
Tip force stiffness [N/m]	$k_{t}$	1	1.18	0.50
Acc. force stiffness [N/m]	$k_{a}$	1.55	2.04	1.17
Deflection at 1 g [Å]	$\Delta x_{tip}$	1.1	0.82	0.9
First natural frequency [kHz]	$f_{1}$	48.1	70.62	59.33
Thermal noise [mg/ √Hz]	TNEA	2.4	2.91	3.4
Lat. stability in x at 1 g [Å]	$w_{x}$	≈0	≈0	0.081
Lat. stability in y at 1 g [Å]	$w_{y}$	0.024	0.019	0.189

**Table 4:** Parameters of the electrostatic actuator.

**Parameter**	**Variable**	**Model 1**		**Model 2**
Actuator length [μm]	$l_{e}$	50		55
Actuator width [μm]	$w_{e}$	50		41
Plate distance [μm]	$d_{e}$	2		2
Initial tip distance [nm]	$d_{t}$	30–300		30–300
Results		analytical	numerical	numerical
Actuator voltage [V]	$U_{e}$	4–12.3	4.6–13.5	4.3–12

**Table 5:** Process steps of fabrication method of small tunneling tips by FIB and GIS.

**Process Step**	**Drawing**	**SEM Image**
1: The initial state shows the untreated PolySi structure with an applied gold pad (top). The left side is connected to the seismic mass and the right side to the bond pad.	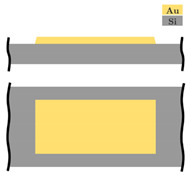	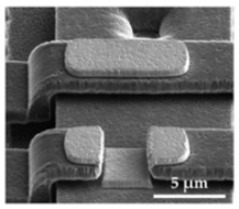
2: In the first processing step, the PolySi structure and the gold pad are structured by a FIB cut with a 260 pA ion current and a width of 1 μm. A small bridge of the PolySi structure remains to keep the spring-mass structure fixed. Next, platinum is deposited by the FIB and the GIS with a $MeCpPtMe_{3}$ precursor.	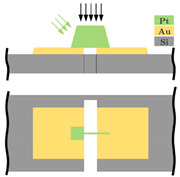	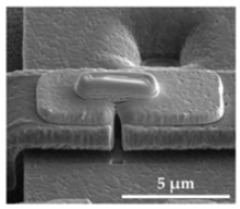
3: To achieve a very thin tip, the platinum gets patterned by the FIB (9 pA to 46 pA). This leads to a vertical nanowire with a length of about 500 nm and a diameter of about 50–100 nm.	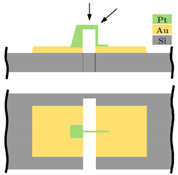	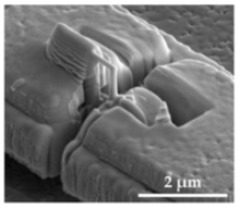
4: With a minimal and sensitive ion current (1.5 pA), the nanowire is further thinned out with a maximum tilt angle of 60° and shaped explicitly into the tip at the separation point. The final step is to release the structure by cutting the still-existing PolySi bridge.	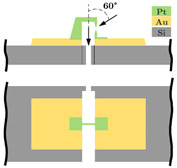	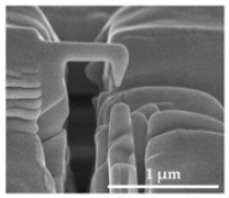

## Data Availability

All data used are shown in the text. Raw data are available on request.

## Abbreviations

The following abbreviations are used in this manuscript.

FIB	Focused Ion Beam
SEM	Scanning Electron Microscope
GIS	Gas Injection System
TEM	Transmission Electron Microscope
Si	Silicon
PolySi	Polysilicon
Pt	Platinum
C	Carbon
PtC	Platinum Carbon
C-H	Carbon-hydrogen
Ga	Gallium
DLC	Diamond Like Carbon
Au	Gold
BMM	Bulk Micro-Machining
SMM	Surface Micro-Machining
SOI	Silicon on Insulator
MEMS	Microelectromechanical System
TNEA	Thermal Noise Equivalent Acceleration
PCB	Printed Circuit Board
SMU	Source Measurement Unit
VdW	Van der Waals
FEM	Finite Element Method
n/a	not available
calc.	calculated
GND	Ground
FFC/FPC	Flexible Flat Cable/ Flexible Printed Circuit
